# Bioinformatic and rare‐variant collapsing analyses for type 1 and type 2 diabetes in the UK Biobank reveal novel pleiotropic susceptibility loci

**DOI:** 10.1111/1753-0407.13452

**Published:** 2023-08-01

**Authors:** Bengt Zöller, Eric Manderstedt, Christina Lind‐Halldén, Christer Halldén

**Affiliations:** ^1^ Center for Primary Health Care Research, Department of Clinical Sciences Lund University and Region Skåne Malmö Sweden; ^2^ Department of Environmental Science and Bioscience Kristianstad University Kristianstad Sweden

Type 1 diabetes (T1D) is a chronic condition caused by the autoimmune destruction of pancreatic β‐cells.^1^ In contrast, type 2 diabetes (T2D) is characterized by impaired glucose metabolism arising from defects in insulin resistance and secretion.^2^ More than 75 genetic loci influencing T1D risk have been identified.^1^ Genome‐wide association studies (GWAS) of T2D have identified over 700 risk loci.^2^ Whole exome sequencing (WES) studies may reveal the association of rare variants to common diseases such as T1D and T2D. However, only a few large‐scale WES studies have been published until Wang et al reported the relationships between rare protein‐coding variants and 17 361 binary phenotypes using WES data from 269 171 UK Biobank participants (https://azphewas.com/).^3^ Recently, Karczewski et al determined gene‐based association investigating 4529 phenotypes in 394 841 UK Biobank exomes (https://app.genebass.org/).^4^

We used the two published UK Biobank portals (https://azphewas.com/ and https://app.genebass.org/)^3^, ^4^ to access gene collapsing analyses of rare variation for T1D and T2D (Table 1). Ethical statements are not required for the study as no human or animal studies are involved. In order not to discard potential candidate genes we present genes with *p* values <.05/20000 genes = 2.5 × 10^−6^ commonly used for WES studies. Identified T1D and T2D genes were bioinformatically analyzed using the GWAS catalog (https://www.ebi.ac.uk/gwas/), OMIM (https://www.omim.org/), and Genecards (https://www.genecards.org/).^5^, ^6^, ^7^, ^8^ The literature was searched for identified genes using https://pubmed.ncbi.nlm.nih.gov/. We compared the union of the same three‐digit ICD‐10 codes (*International Classification of Diseases, Tenth Revision*).^3^, ^4^ In Table 1 only the genes with genome wide significant results are shown with *p* values for the most significant model. One previously T1D linked gene (*HLA‐DRB5*) and four novel T1D genes (*PSMB9*, *NELFE*, *SLC44A4*, and *VWA7* ) were identified. For T2D four previously linked genes ( *GCK* , *HNF1A* , *HNF4A* , and *ANKH* ) were confirmed. In addition, *GIGYF1* has recently already been linked to T2D in UK Biobank.^9^ Two novel associations were identified, the *DENND6A* and *RPS3A* genes. The identified genes were specific for each of T1D and T2D (Table 1).

**TABLE 1:** Results of gene collapsing analysis of rare variants for type 1 diabetes (T1D) and type 2 diabetes (T2D) according to ICD‐10 codes (https://azphewas.com/ and https://app.genebass.org/).^3^, ^4^

ICD‐10 codes	Genebass (LOF)	Genebass (missense/LC)	Astra Zeneca portal
E10 T1D	NS	*PSMB9* 1.59 × 10^−9^	* *HLA‐DRB5* 5.70 × 10^−7^
		*NELFE* 1.74 × 10^−9^	*VWA7* 1.99 × 10^−6^
		*SLC44A4* 1.85 × 10^−9^	
E11 T2D	** *GCK* 5.86 × 10^−20^	** *ANKH* 1.32 × 10^−12^	** *GCK* 8.18 × 10^−21^
	*GIGYF1* 1.55 × 10^−11^	** *GCK* 4.43 × 10^−8^	*GIGYF1* 9.98 × 10^−11^
	** *HNF1A* 4.59 × 10^−7^	*DENND6A* 5.12 × 10^−7^	** *HNF1A* 7.13 × 10^−8^
		** *HNF4A* 1.74 × 10^−6^	*RPS3A* 2.05 × 10^−6^
PheWAS for identified T1D genes			
*PSMB9*	NS	Ankylosing spondylitis 3.77 × 10^−8^	NS
		H20 Iridocyclitis 5 × 10^−8^	
*NELFE*	NS	M45 Ankylosing spondylitis 3.44 × 10^−13^	NS
		H20 Iridocyclitis 9 8.05 × 10^−14^	
		Iritis 2.66 × 10^−7^	
*SLC44A4*	NS	M45 Ankylosing spondylitis 1.72 × 10^−20^	NS
		H20 Iridocyclitis 1.48 × 10^−13^	
* *HLA‐DRB5*	NS	E03 Other hypothyroidism 1.15 × 10^−6^	J45 Asthma 7.69 × 10^−10^
			K90.0 Coeliac disease 1.03 × 10^−9^
			Sarcoidosis 1.32 × 10^−7^
*VWA7*	NS	NS	K90.0 Coeliac disease 5.34 × 10^−14^
			L40 Psoriasis 1.58 × 10^−10^
			Rheumatoid arthritis 5.96 × 10^−7^
			D41 Neoplasm of uncertain or unknown behavior of urinary organs 1.84 × 10^−6^
			I10‐I15 Hypertensive diseases 2.38 × 10^−6^
PheWAS for identified T2D genes			
** *GCK*	NS	NS	H36 Retinal disorders in diseases classified elsewhere 2.86 × 10^−8^
			Chapter IV Endocrine|nutritional and metabolic diseases 9.41 × 10^−7^
*GIGYF1*	E03 Other hypothyroidism 1.2 × 10^−7^	NS	E03 Other hypothyroidism 7.12 × 10^−9^
	J44 Other chronic obstructive pulmonary disease 1.57 × 10^−6^		E00‐E07 Disorders of thyroid gland 3.47 × 10^−8^
			E03.9 Hypothyroidism| unspecified 3.84 × 10^−8^
			Hypothyroidism|myxoedema 1.45 × 10^−6^
			R55 Syncope and collapse 2.26 × 10^−6^
** *HNF1A*	NS	NS	NS
** *ANKH*	NS	NS	NS
*DENND6A*	NS	NS	NS
** *HNF4A*	NS	NS	NS
*RPS3A*	ND	NS	NS

*Note*: Union was used to define phenotypes for https://azphewas.com. Only genome‐wide significant associated genes are shown (for the most significant model) that is, *p* < 2.5 × 10^−6^. Other disease phenotypes than diabetes were searched in the databases for the identified T1D and T2D genes, that is phenome‐wide association study (PheWAS). Significance threshold was *p* value <.05/20000 genes = 2.5 × 10^−6^ commonly used for WES studies. For Genebass portal (https://app.genebass.org the most significant *p* value for SKAT, SKAT‐O, or burden test is shown.^5^ For Astra Zeneca portal (https://azphewas.com) the most significant *p* value of the 12 tested models is shown.^3^ ICD‐10, *International Classification of Diseases, Tenth Revision*; LoF, high‐confidence loss of function variants indicated by LOFTEE^4^; Missense/LC, Missense variants are grouped with in‐frame insertions and deletions, as well as low‐confidence LoF variants filtered out by LOFTEE^4^ (The latter have a frequency spectrum consistent with missense variation and affect a set of amino acids in a similar way^4^); NS, no genome wide significant gene; ND, not determined; SKAT, sequence kernel association test; SKAT‐O, optimized sequence kernel association test; WES, whole exome sequencing.

* The *HLA‐DRB5* locus has been linked to T1D in genome‐wide association study (GWAS) (https://www.ebi.ac.uk/gwas/).

** The *ANKH, GCK, HNF1A*, and *HNF4A* loci have been linked to T2D in GWAS (https://www.ebi.ac.uk/gwas/).

Phenome‐wide association studies (PheWAS) data (Table 1) could link all five identified T1D genes to other immune‐mediated diseases: ankylosing spondylitis, iridocyclitis, hypothyroidism, asthma, celiac disease, sarcoidosis, psoriasis, and rheumatoid arthritis (Table 1). Thus, the five identified pleiotropic T1D genes may all contribute to the previously observed epidemiological associations between T1D and other immune‐mediated diseases.^10^ Only the *GIGYF1* gene among the T2D linked genes was associated with a potential immune‐mediated disorder (hypothyroidism) (Table 1). However, even more interesting is the association between the T2D linked *GIGYF1* gene and chronic obstructive pulmonary disease (COPD) in the PheWAS analysis (Table 1). COPD and T2D are recognized to be associated conditions with shared environmental exposures.^11^ Treatment with the novel antihyperglycemic drugs glucagon‐like peptide 1 (receptor agonists and sodium glucose transporter 2 inhibitors have recently been associated with a reduced risk of severe exacerbations in COPD among patients with T2D.^12^ Thus, the *GIGYF1* gene might contribute to the observed epidemiological association between COPD and T2D and may open novel treatments for T2D and COPD.

A limitation is that the validity of T1D is not perfect in UK Biobank. However, a diagnosis of T1D in UK Biobank may still be useful for research in large studies. Two papers about T1D in UK Biobank have been published suggesting that the UK Biobank might be useful for T1D research: one in *Lancet Diabetes & Endocrinology* and one in *Diabetes Medicine*.^13^, ^14^ Moreover, an article by Thomas et al has shown that the accuracy of T1D and T2D tested with two different methods range from 71% to 88%.^15^ These articles are in line with the findings in the present study. For instance, we confirmed one previously recognized T1D gene (*HLA‐DRB5*) and four previously linked T2D genes ( *GCK* , *HNF1A* , *HNF4A* , and *ANKH* ). Thus, the definition used in UK Biobank for T1D and T2D could differentiate between known T1D and T2D genes, which is reassuring. Moreover, all the T1D linked genes (one old and four novel T1D genes) could in the PheWAS and the bioinformatic analysis be linked to immune‐mediated disorders (Tables 1 and 2). It is well known that genetic links between many different immune‐mediated disorders exist.^10^ Moreover, no T1D gene was linked to T2D. Thus, there was no overlap between T1D and T2D genes. Moreover, only one T2D gene could be linked in the PheWAS and bioinformatic analysis to a potential immune‐mediated condition (ie, hypothyroidism). Thus, we believe the accuracy is acceptable for large studies of T1D and T2D genetics in UK Biobank.

**TABLE 2:** Bioinformatic analysis of associated with type 1 diabetes (T1D) and type 2 diabetes (T2D) according to ICD‐10 codes in the genes collapsing analysis of rare variants in the two published UK Biobank portals (https://azphewas.com/ and https://app.genebass.org/).^3^, ^4^

Genes	GWAS catalog*	OMIM/genecards*	Pathway analysis*
Type 1 diabetes			
*PSMB9c*	Rheumatoid arthritis (RA), autoimmune disease	Proteasome‐associated auto inflammatory syndrome	Regulation of activated PAK‐2p34
*NELFE*	Age‐related macular degeneration	Immunodeficiency	Formation of HIV elongation complex
*SLC44A4*	Systemic lupus erythematosus (SLE), vitiligo	Deafness	Transport of cations/anions/amino acids/peptides
*HLA‐DRB5*	T1D, asthma, nephrotic syndrome, RA, SLE	Pityriasis rosea, Parkinson disease	TCR signaling (Qiagen)
*VWA7*	Takayasu arteritis, asthma, Crohn prognosis	Armfield syndrome	‐
Type 2 diabetes			
*GCK*	T2D, metabolic syndrome	Maturity‐onset diabetes of the young	Glycolysis and trehalose degradation
*GIGYF1*	Autoimmune thyroid disease, hypothyroidism	Parkinson disease	‐
*HNF1Ac*	T2D	Maturity‐onset diabetes of the young	Regulation of beta‐cell development
*ANKH*	T2D	Craniometaphyseal dysplasia, chondrocalcinosis	Transport of cations/anions/amino acids/peptides
*DENND6A*	Alzheimer disease	‐	Vesicle‐mediated transport, Rab trafficking
*HNF4A*	T2D	Maturity‐onset diabetes of the young	Gene transcription, nuclear receptors
*RPS3A*	‐	Diamond‐Blackfan anemia, encephalopathy	Peptide chain elongation

*Note*: The GWAS catalog (https://www.ebi.ac.uk/gwas/),^5^ OMIM (https://www.omim.org/),^6^ and Genecards (https://www.genecards.org/)^7^, ^8^ were searched for the association with relevant diseases and pathways.*

Abbreviations: GWAS, genome‐wide association study; ICD‐10, *International Classification of Diseases, Tenth Revision*; OMIM, Online Mendelian Inheritance in Man; TCR, T cell receptor.

* The most potentially relevant diseases and pathways are shown. For a complete list the reader is referred to the original bioinformatic resources: that is, GWAS catalog (https://www.ebi.ac.uk/gwas/), OMIM (https://www.omim.org/), and Genecards (https://www.genecards.org/).

In conclusion, rare variations in 12 genes (six novel) were associated with diabetes in the UK Biobank, T1D (five genes) and T2D (seven genes). Thus, rare variation contributes to T1D and T2D in the general population. Rare variation in all five T1D linked genes is also linked to other immune‐mediated diseases in UK Biobank, whereas the T2D gene *GIGYF1* is associated with COPD.
